# An innovative method to assess clinical reasoning skills: Clinical reasoning tests in the second national medical science Olympiad in Iran

**DOI:** 10.1186/1756-0500-4-418

**Published:** 2011-10-17

**Authors:** Mitra Amini, Mohsen Moghadami, Javad Kojuri, Hamidreza Abbasi, Ali Arhami Doolat Abadi, Nezar Ali Molaee, Elham Pishbin, Hamid Reza Javadzade, Vahid Monsef Kasmaee, Hasan Vakili, Mohamad Ali Reis Sadat, Roghaye Akbari, Bita Omidvar, Afshin Shafaghi, Marzie Dehbozorgian, Mohammad Morad Jafari, Alireza Monajemi, Kamran Soltani Arabshahi, Peyman Adibi, Bernard Charlin

**Affiliations:** 1 Education Development and Research Center, Shiraz University of Medical Sciences, Shiraz, Iran; 2 Emam Hosien Hospital Emergency Department, Shahid Beheshti University of Medical Sciences, Tehran, Iran; 3 Internal Medicine Aliebneabitaleb Hospital, Zahedan University of Medical Sciences, Zahedan, Iran; 4 Emam Reza Emergency Medicine Department, Mashhad University of Medical Sciences, Mashhad, Iran; 5 Emergency Medicine Department, Baghiatallah Hospital, Baghiatallah University of Medical Sciences, Tehran, Iran; 6 Department of Emergency Medicine, Guilan University of Medical Science, Rasht, Iran; 7 Internal Medicine Department, Babol University of Medical Sciences, Babol, Iran; 8 Pediatric General Surgery, Islamic Azad University, Mashhad Branch, Mashhad, Iran; 9 Internal Medicine Department, Ahwaz Hospital, Ahwaz University of Medical Sciences, Ahwaz, Iran; 10 Medical Humanities Research Group, Tehran, Institute for Humanities and Cultural Studies, Tehran, Iran; 11 Firozgar Hospital of Internal Medicine, Tehran University of Medical Sciences, Tehran, Iran; 12 Integrative Functional Gastroenterology Research Centers, Isfahan University of Medical Sciences, Isfahan, and Head of the Olympiad Technical Committee, Ministry of Health and Medical Education, Tehran, Iran; 13 Recherche et Développement, Centre de Pédagogie Appliquée aux Sciences de la Santé, Montreal University, Montreal, Canada

## Abstract

**Background:**

Clinical reasoning plays a major role in the ability of doctors to make a diagnosis and reach treatment decisions. This paper describes the use of four clinical reasoning tests in the second National Medical Science Olympiad in Iran: key features (KF), script concordance (SCT), clinical reasoning problems (CRP) and comprehensive integrative puzzles (CIP). The purpose of the study was to design a multi instrument for multiple roles approach in clinical reasoning field based on the theoretical framework, KF was used to measure data gathering, CRP was used to measure hypothesis formation, SCT and CIP were used to measure hypothesis evaluation and investigating the combined use of these tests in the Olympiad. A bank of clinical reasoning test items was developed for emergency medicine by a scientific expert committee representing all the medical schools in the country. These items were pretested by a reference group and the results were analyzed to select items that could be omitted. Then 135 top-ranked medical students from 45 medical universities in Iran participated in the clinical domain of the Olympiad. The reliability of each test was calculated by Cronbach's alpha. Item difficulty and the correlation between each item and the total score were measured. The correlation between the students' final grade and each of the clinical reasoning tests was calculated, as was the correlation between final grades and another measure of knowledge, i.e., the students' grade point average.

**Results:**

The combined reliability for all four clinical reasoning tests was 0.91. Of the four clinical reasoning tests we compared, reliability was highest for CIP (0.91). The reliability was 0.83 for KF, 0.78 for SCT and 0.71 for CRP. Most of the tests had an acceptable item difficulty level between 0.2 and 0.8. The correlation between the score for each item and the total test score for each of the four tests was positive. The correlations between scores for each test and total score were highest for KF and CIP. The correlation between scores for each test and grade point average was low to intermediate for all four of the tests.

**Conclusion:**

The combination of these four clinical reasoning tests is a reliable evaluation tool that can be implemented to assess clinical reasoning skills in talented undergraduate medical students, however these data may not generalizable to whole medical students population. The CIP and KF tests showed the greatest potential to measure clinical reasoning skills. Grade point averages did not necessarily predict performance in the clinical domain of the national competitive examination for medical school students.

## Background

Clinical reasoning is defined as the process by which information about a clinical problem is combined with the previous physicians' knowledge and experiences and used to manage a particular problem [1]. This process is an important factor in the physician's competence. Educators agree that clinical reasoning should be taught and tested in medical schools [2]. Attempts to assess clinical reasoning began in the 1970s [3]. The most popular test was the patient management problem(PMP) instrument developed at the University of Illinois [1]. This device is a test of clinical problem-solving skills in which each item begins with a clinical statement about the patient's problems on presentation. It is structured in stages, and the examinee is asked to make a decision that is appropriate to the situation [4,5]. Due to the long duration of PMP and its low reliability, other clinical reasoning tests were introduced, such as the key features (KF) test described in 1987 [6]. A KF is defined as a critical step in the resolution of a problem [7]. Testing with this instrument for the Canadian Qualifying Examination in medicine was shown to have an acceptable content validity and a reliability of 0.8 in 4 hours of examination time [8].

The script concordance test (SCT) is a clinical reasoning test used to assess reasoning in ambiguous situations [9]. This test is case-based, and items describe short scenarios followed by a series of questions presented in three parts. Part one contains a relevant diagnostic or management option, part two presents a new clinical finding and part three is a five-point Likert scale from - 2 to +2 that indicates examinees' decisions [10]. The SCT is based on the principle that concordance can be measured between the examinees' answers and a panel of experts' judgments [11]. Research on the reliability and validity of SCT showed they are content valid, reliable and linearly related to experience [12-15], however more research is required to evaluate differential validity compared to multiple choice questions and predictive validity linked to clinical performance [16]

Another test of clinical reasoning is the set of clinical reasoning problems (CRP). In these items a scenario is presented and examinees are asked to nominate the two diagnoses they consider most likely, list the features of the case they think are important for the diagnosis, and indicate whether these features are positively or negatively predictive. Few studies was done on validity and reliability of CRPs but results showed an improved estimate of validity and reliability, especially proving the usefulness of CRPs as an indicator of the accuracy of the diagnostic reasoning [17].

Another assessment method is the comprehensive integrative puzzle (CIP). Items in this test are presented in the format of an extended matrix of rows and columns, in which examinees must insert the correct information in each cell. This test measures diagnostic thinking and clinical reasoning, However, this instrument seems to appeal more to students because of the fun in solving matching puzzles [18].

Research on clinical reasoning is scattered throughout medical education journals or publications in other field such as cognitive psychology and clinical psychology. Each of the tests described above was introduced in different studies, and to our knowledge different kinds of clinical reasoning tests have not been systematically studied or compared. The purpose of our study was to design a multi instrument for multiple roles approach in clinical reasoning field based on the theoretical framework, KF was used to measure data gathering, CRP was used to measure hypothesis formation, SCT and CIP were used to measure hypothesis evaluation and investigating the combined use of these tests in a single, nationwide, comprehensive, competitive examination for medical students known as the National Medical Science Olympiad. We examined the correlation between the total examination score and scores on each of four clinical reasoning tests. We also searched for a possible correlation between the total examination score and another measure of knowledge (grade point average).

## Methods

The main aim of the Medical Science Olympiad in Iran is to test creative and critical thinking in medical students. The specific objectives of Olympiad were: Identifying scientifically talented individuals, Motivating and encouraging scientifically talented Individuals, Orienting extra-circular scientific activities, Generating scientific liveliness and morale, Interuniversity cultural exchanges, Encouraging to creative and critical thinking, Reinforcing health system goals and objectives, Encouraging team work, Encouraging interdisciplinary activities [19].

The first Olympiad, held in Isfahan in 2009, and the second in Shiraz in 2010, comprised a separate examination in each of three areas: basic science, clinical science and health system management. All currently enrolled medical students with a grade point average of 16/20 (equivalent to a GPA of about 3.2 in the USA or a UK Class of about 60) or higher were eligible to register for the test. Then they prepared for the test by completing an intensive training course in the area of their choice at their own university. After this course enrollees were tested for critical thinking and reasoning skills at their university, and only those with the highest grades were then allowed to participate in the national Olympiad. Iran has 46 medical universities and each university is allowed to send only 3 students in each of the three areas to the Olympiad.

In the second Olympiad, 45 medical universities sent examinees for the areas of basic science and clinical science, and 44 medical universities sent examinees for the area of health system management. A total of 135 students took the test in basic science, 135 students were tested in clinical science, and 131 students took the test for the management area. In this study we analyzed the results only for the examination in the clinical science area. Only undergraduate students were allowed to participate in the Olympiad because of the importance of clinical reasoning skills in an early stage of their medical education and the need for efficient tools to assess it.

### Development of the clinical reasoning tests

An expert committee with members from all Iranian medical schools was constituted and charged with developing a bank of test items in emergency medicine from all four clinical reasoning tests (i.e., KF, SCT CRP and CIP). The committee used the methodology described in previous publications [6-18]. Some examples of these tests are provided in additional file 1.

### Development of the Olympiad examination by the reference panel

To prepare the examination to be used in the Olympiad, a total of 15 experts from different medical universities in Iran were chosen to constitute the reference panel. These experts comprised a broad sample of internists, general surgeons and emergency medicine specialists with different levels of experience and training, and were therefore considered to represent a normative sample of the reference population. Each member of the reference panel took each of the four tests and identified test items that were confusing or not relevant to emergency medicine. As a result, a few minor changes were made in the wording of some items. Then 20 KF items, 20 SCT items,10 CRP items and two 4 × 6 matrices from the CIP were chosen for inclusion in the full 2-day Olympiad examination. On the morning of the first day the 20 KF items were completed, and in the afternoon the 10 CRP items were completed. On the morning of the second day the 20 SCT items were completed, and in the afternoon the two CIP matrices were completed. Each of the four examination periods lasted 4 hours.

### Examinees

The examinees in the second Olympiad were 135 undergraduate medical students from 45 medical schools in Iran, with grade point average if 16/20 or higher. The length of medical education in Iran is 7 years.57.8 percent of participants were females and 42.2 percents were males. The mean year of study of participants was 6.1 years, the mean age of them was 24,3 years and the mean grade point average of them were 17.6 from 20.

### Scoring process

A group of 22 general practitioners and first-year residents were asked to complete all Olympiad examination items in their own time without using textbooks, web sites or personal consultations. General practitioners and first-year residents were recruited for this group because of their experience with a wide range of clinical problems encompassing all areas of emergency medicine practice. The scores obtained by these examinees were used as a standard reference [20].

### KF scoring

To enhance the discriminating power of this score, we also calculated the efficiency score (partial credit score) [8].

### SCT scoring

For high-stakes SCT examinations a reference group of more than 20 members is required [21]; as noted above, our reference group consisted of 22 physicians. Because of issues with aggregated scoring such as greater random error [16], we used average expert response weighted for distance and the correct answer on a five-point Likert scale. The mean response was considered the correct answer, and the weight for other responses was determined based on their credit and distance from the correct answer. With this scoring system the credit for the best answer was 100%, and credit for other answers was calculated based on the percentage of reference panel examinees who chose that answer. We used the formula 1/ (1 + *x*), where *x *is defined as the distance from the correct answer (values of *x *ranged from a minimum of 1 to a maximum of 4). This innovative scoring system was devised in the light of an analysis by Bland et al. [16] and consultation with a mathematician familiar with that research.

### CRP scoring

The first and second diagnoses and diagnostic features chosen for each item by reference group examinees were input into a table, and the diagnoses and nominated features that were chosen by at least two thirds of the reference group were considered the correct answers.

### CIP scoring

Examinees' scores were calculated from a matrix of answers given by the reference panel. For each of the 4 columns of cells in the matrix, 4 correct answers out of 4 (4/4) was scored as 100%, 3/4 as 75%, 2/4 as 50% and 1/4 as 0%. The grade for an entire matrix was considered the sum of the grades for all six rows and the grade for CIP exam was measured by the sum of two matrix grades.

#### Total exam scores

The total exam score was measured by the sum of 4 tests grade, therefore each test counts 25 percent of the total grade. The expert committee believed that 10 CRPs is similar to 20 KFs or 20 SCT because in CRP the students should choose two diagnoses and list the features of case based on these two diagnoses. In the CIP due to complexity of puzzles the expert committee considered two 6*4 puzzle similar to 20 KFs or 20 CRPs. As we mentioned earlier the similar exam time was considered for each of the four tests(four hour for each tests).

### Analysis

We measured item difficulty for each test, and determined the reliability of the scoring method for each test. The reliability of each test was calculated with Cronbach's alpha, considering each item individually and the combined reliability for all four clinical reasoning tests was calculated using variances of score in each test and total exam variance [22]. Item difficulty was determined with the method of Whitney and Sabers [23], and correlations between the total examination score and scores for each item were calculated with Pearson's correlation coefficient for each of the four clinical reasoning tests. The correlation between the total score and scores on each of the four tests was also calculated, along with the correlation between the total score on the Olympiad and the student's university course grade point average. We sought an informed consent from participants and ethical approval for our study from Olympiad clinical domain.

## Results

The scores of each test in the Olympiad, total Olympiad score and maximum and minimum of each scor are shown in table 1. The reliability of KF was .83, and this measure was .78 for SCT,.71 for CRP and .91 for CIP. The combined reliability for all four clinical reasoning tests was 0.91.

**Table 1:** Olympiad examination Scores

	KF score (from 500)	SCT score (from 500)	CRP score (from 500)	CIP score (from 500)	Total Olympiad score (from 2000)
Mean score ± SD	284.06 ± 40.39	217.66 ± 40.30	166.19 ± 36.14	258.58 ± 84.84	1060.84 ± 201.56

Maximum	353	316	302	378	1643

Minimum	150	112	81	22	566.5

Our findings for item difficulty level and item-total correlation are summarized for all 20 KF items in Table 2, all 20 SCT items in Table 3, all 10 CRP items in Table 4, and the two 6 × 4 matrices in Table 5. An item difficulty level between 0.2 and 0.8 is recommended [24] to differentiate between high- and low-achieving students, and the item-total correlations should be positive. In the KF test, item difficulty for all 20 items was between 0.52 and 0.79. For the CRP test, all 10 items had a difficulty index between 0.40 and 0.69. In the SCT (the most difficult of the four tests), item difficulty was between 0.25 and to 0.57 for 19 of the 20 items, but was 0.15 for one of the items. Item difficulty in the CIP test ranged from 0.39 to 0.90.

**Table 2:** Item difficulty level and item-total correlations for the Key Features (KF) test.

	KF1	KF2	KF3	KF4	KF5	KF6	KF7	KF8	KF9	KF10
Item difficulty level	0.52	0.66	0.66	0.76	0.77	0.69	0.69	0.79	0.71	0.71

Item-total correlation	0.51	0.38	0.53	0.30	0.33	0.59	0.50	0.37	0.39	0.28

	**KF11**	**KF12**	**KF13**	**KF14**	**KF15**	**KF16**	**KF17**	**KF18**	**KF19**	**KF20**

Item difficulty level	0.70	0.74	0.72	0.66	0.78	0.57	0.64	0.61	0.76	0.53

Item-total correlation	0.33	0.37	0.38	0.49	0.49	0.59	0.14	0.56	0.54	0.49

**Table 3:** Item difficulty level and item-total correlations for the Script Concordance Test (SCT)

	SC1	SC2	SC3	SC4	SC5	SC6	SC7	SC8	SC9	SC10
Item difficulty level	0.36	0.52	0.25	0.60	0.48	0.44	0.43	0.42	0.57	0.48

Item-total correlation	0.41	0.46	0.32	0.45	0.50	0.37	0.52	0.48	0.33	0.41

	**SC11**	**SC12**	**SC13**	**SC14**	**SC15**	**SC16**	**SC17**	**SC18**	**SC19**	**SC20**

Item difficulty level	0.49	0.40	0.43	0.24	0.51	0.32	0.49	0.48	0.15	0.21

Item-total correlation	0.54	0.41	0.30	0.19	0.30	0.34	0.28	0.41	0.12	0.09

**Table 4:** Item difficulty level and item-total correlations for the Clinical Reasoning Problems (CRP) test

	CRP1	CRP2	CRP3	CRP4	CRP5	CRP6	CRP7	CRP8	CRP9	CRP10
Item difficulty level	0.57	0.51	0.69	0.54	0.40	0.56	0.43	0.64	0.49	0.53

Item-total correlation	0.61	0.39	0.44	0.44	0.50	0.51	0.49	0.32	0.49	0.52

**Table 5:** Item difficulty level and item-total correlations for the Comprehensive Integrative Puzzles (CIP) test

	Q1	Q2	Q3	Q4	Q5	Q6	Q7	Q8	Q9
Item difficulty level	0.90	0.56	0.56	0.59	0.69	0.56	0.39	0.55	0.57

Item-total correlation	0.36	0.67	0.69	0.53	0.66	0.51	0.50	0.69	0.66

	**Q10**	**Q11**	**Q12**	**Q13**	**Q14**	**Q15**	**Q16**	**Q17**	**Q18**

Item difficulty level	0.50	0.50	0.51	0.67	0.60	0.57	0.72	0.68	0.66

Item-total correlation	0.55	0.67	0.59	0.77	0.64	0.66	0.78	0.77	0.80

All item-total correlations were positive. The correlations between each of the clinical reasoning test scores and the total examination score were high, although the highest correlations were seen for the KF and CIP tests (Table 6).

**Table 6:** Correlations between total Olympiad examination score and scores on each of the four clinical reasoning tests

Correlation coefficient	Key Features (KF)	Clinical Reasoning Problems (CRP)	Script Concordance Test (SCT)	Comprehensive Integrative Puzzle (CIP)
Total grade	0.80	0.74	0.67	0.77

Significance (P value)	<0.001	<0.001	<0.001	<0.001

The correlation between scores on each clinical reasoning test and students' grade point average was low to intermediate for different tests (Table 7).

**Table 7:** Correlations between grade point average and scores on each of the four clinical reasoning tests

Correlation coefficient	Key Features (KF)	Clinical Reasoning Problems (CRP)	Script Concordance Test (SCT)	Comprehensive Integrative Puzzle (CIP)
Grade point average	0.26	0.27	0.13	0.18

Significance (P value)	<0.01	<0.01	>0.10	<0.05

## Discussion

The purpose of this study was to investigate the combined use of four different clinical reasoning tests (KF, SCT, CRP and CIP) in a high-stakes national examination designed to test clinical reasoning and decision-making skills in medical school undergraduates. Our results showed that the reliability of all four clinical reasoning tests was high. The most reliable tests were the CIP followed by the KF test, whereas the reliability of the SCT and CRP test was lower.

Different studies have reported varying reliabilities for these tests. The reliability for the KF test has been variously reported as 0.49 [25], 0.65 [26] and 0.80 in a 4-hour examination [8]. Our partial credit scoring approach for this test led to more reliable results than in other studies. Although few studies have focused on the CRP, earlier reliability values ranged from 0.61 to 0.83 [17], which were similar to the reliability values we found. In the present study the reliability of the SCT was 0.78. The psychometric properties of five scoring methods applied to the SCT were determined by Bland and et al. [16]. The reliability of these scoring methods ranged from 0.68 to 0.78. Bland and colleagues reported that single-best-answer scoring with three answer choices produced results similar to aggregate scoring on a Likert-type scale, although they concluded that the optimal SCT scoring process is still debated [16]. In the present study the expert committee that chose the items for inclusion in the Olympiad examination believed that three answer choices increased the probability of choosing the answer by chance. The average expert response weighted for distance with our innovative formula and the correct answer from a five-point Likert scale showed acceptable reliability, although further research is necessary to compare this method with previous scoring methods.

With regard to item difficulty, except for item number 19 on the SCT, all other test items had an acceptable level of difficulty between 0.20 to 0.80. In terms of item-total correlations, all correlations were positive although for a few items this correlation was poor. In general, the findings for these correlations showed KF item 17 and SCT items 14, 19 and 20 were not able to discriminate effectively between high-achieving and low-achieving participants.

Our positive results, together with content validation of the tests before the Olympiad, enhanced the validity of the four-part, two-day examination. High correlation between each of the clinical reasoning tests and total Olympiad grade was an indicator for concurrent validity of these tests and also construct validity of the whole examination. However, the correlations between the clinical reasoning test results and grade point averages was low to intermediate, a finding that supports the idea that routine examination at medical universities in our setting measures students' factual knowledge more than their clinical reasoning skills.

Among the most important strengths of the present study is the large sample of examinees from all medical universities in Iran. Moreover, we used an expert panel of teachers from different medical universities to screen, select and adapt the items from all four tests that they felt were mostly likely to yield accurate results. The main limitation of our study was that examinees were strictly screened and selected from among the best students at each medical university. Olympiad scores cannot be viewed as generalizable to the whole population of medical students. This restriction of range may actually have enhanced the finding especially item difficulty level and correlations if all students had participated. Other limitations of our study were the facts that we did not compare different scoring methods, the pen-and-pencil format of the Olympiad, and the manual scoring of the Olympiad examinations. Some of these strengths and limitations are reported in results of the first Olympiad in Isfahan too [27]. This indicate the need to improve technical elements of the examination such as computerized administration and scoring. Future studies should be designed to validate our examination design and assessment methods.

## Conclusion

We hope that the combination of clinical reasoning tests we used in a high-stakes national level examination for medical school undergraduates will provide evidence to support future actions aimed at enhancing the reliability of this exercise. We further hope that this report will help to raise the important issue of test reliability and motivate other universities and medical schools in other settings to examine their testing policies.

## Competing interests

The authors declare that they have no competing interests.

## Authors' contributions

MA-MM-JK coordinated the team, collected the data, contributed to data analysis and interpretation, and drafted the manuscript. AM - KSA- PA contributed to the conception of clinical reasoning battery in Olympiad, contributed to design of the study and supervised the whole study. HA-AAD-NAM-EP-HJ-VMK-HV-MARS-RA-BO-AS-MD-MM contributed to the design of the various tests and involved in scoring them. BC commented on the draft at all stages. All authors read and approved the final manuscript.

## Supplementary Material

Additional file 1 **Appendix**. A sample of clinical reasoning tests Click here for file

## References

[B1] Newble D Norma G Van der Vulten CP Jones JHM Assessing clinical reasoning in Clinical reasoning in health professions 2 156 188

[B2] Van der Vulten CPM Newble DI How can we test clinical reasoning? Lancet 1995 345 1032 1034 10.1016/S0140-6736(95)90763-7 7605439

[B3] Norman G Research in clinical reasoning: Past history and current trends Medical Education 2005 39 418 427 10.1111/j.1365-2929.2005.02127.x 15813765

[B4] Harden RM Preparation and presentation of patient management problems (PMPS) Medical Education 1983 17 4 255 276 10.1111/j.1365-2923.1983.tb01459.x 6192317

[B5] Marshall JR Fabb WE The construction of patient management problems Medical Education 1981 15 2 126 135 10.1111/j.1365-2923.1981.tb02410.x 7207273

[B6] Bordage G Page G Hart I, Harden R An alternate approach to PMPS, the key feature concept Further developments in assessing clinical competent Montreal 1987 Can Heal Publications 57 75

[B7] Farmer EA Page G A practical guide to assessing clinical decision making skills using the key feature approach Medical Education 2005 39 1188 1194 10.1111/j.1365-2929.2005.02339.x 16313577

[B8] Page G Bordage G The medical council of Canada's key feature project: a more valid written examination of clinical decision making sills Acad Med 1995 70 104 110 10.1097/00001888-199502000-00012 7865034

[B9] Fournier JP Demeester A Charlin B Script Concordance tests: guideline for constriction BMC medical informatics and decision making 2008 8 18 1 7 18460199 10.1186/1472-6947-8-18 PMC2427021

[B10] Charlin B Boshuizen HPA Custers EJFM Feltovich PJ Scripts and clinical reasoning Medical Education 2007 41 1179 1185 10.1111/j.1365-2923.2007.02924.x 18045370

[B11] Charlin B Vander ulten CPM Standardized assessment of reasoning in context of uncertainty. The script concordance test approach Evaluation and the Health Profession 2004 27 304 319 10.1177/0163278704267043 15312287

[B12] Brailovsky C Charlin B Beausoleil S Cote S Van der Vleuten C Measurement of clinical reflective capacity early in training as a predictor of clinical reasoning performance at the end of residency: an exploratory study on the script concordance test Med Educ 2001 35 430 6 10.1046/j.1365-2923.2001.00911.x 11328512

[B13] Charlin B Brailovsky CA Brazeau-Lamontague L Samson L Leduc C Script questionnaires: their use for assessment of diagnostic knowledge in radiology Med Teach 1998 20 567 71 10.1080/01421599880300

[B14] Charlin B Brailovsky CA Leduc C Blouin D The diagnostic script questionnaire: a new tool to assess a specific dimension of clinical competence Adv Health Sci Educ 1998 3 51 8 10.1023/A:1009741430850 12386395

[B15] Charlin B Brailovsky C Roy L Goulet F van der Vleuten C The script concordance test: a tool to assess the reflective clinician Teach Learn Med 2000 12 189 95 10.1207/S15328015TLM1204_5 11273368

[B16] Bland AC Clarence D Kreiter CD Gordon JA The psychometric properties of five scoring methods applied to the script concordance test Academic Medicine 2005 80 4 395 9 10.1097/00001888-200504000-00019 15793026

[B17] Groves M Scott I Alexander H Assessing clinical reasoning: a method to monitor its development in a PBL curriculum Medical Teacher 2002 24 5 507 515 10.1080/01421590220145743 12450471

[B18] Ber R The CIP (comprehensive Integrative puzzle) assessment method Medical Teacher 2003 25 2 171 176 10.1080/0142159031000092571 12745526

[B19] Amini M Kojuri J Karimian Z Lotfi F Moghadami M Dehghani MR Talents for future: Report of the second national medical science Olympiad in Islamic republic of Iran Iran Red Crescent Med J 2011 13 6 377 381

[B20] Norcini J Shea JA Day SC The use of the aggregated scoring for a recertification examination Eval Health Prof 1990 13 241 251 10.1177/016327879001300207

[B21] Gagnon R Charlin B Coletti M Sauve E Van der vulten CPM Assessment in the context of uncertainty: How many members are needed on the panel of reference of a script concordance test? Medical Education 2005 39 284 291 10.1111/j.1365-2929.2005.02092.x 15733164

[B22] Carmines EG Zeller RA Relability and validity assessment Sage 1997

[B23] Whitney DR Sabers DL Improving essay examinations III: Use of item analysis 1970 Iowa City: University of Iowa, University Evaluation and Examination Service (Technical Bulletin No. 11)

[B24] Bortz J Do Ring N Forschungs methoden und Evaluation 2002 3 Berlin, Springer

[B25] Hatala R Norman G Adapting the key features examination for a clinical clerkship Medical Education 2002 36 160 165 10.1046/j.1365-2923.2002.01067.x 11869444

[B26] Fischer MR Kopp V Holzer M Ruderich F Junger J A modified electronic key feature examination for undergraduate medical students: validation threats and opportunities Medical Teacher 2005 27 5 450 455 10.1080/01421590500078471 16147800

[B27] Monajemi A Adibi P Soltani Arabshahi K Arbabi F Akbari R Custers E HadadgaR A Hadizadeh F Changiz T The battery for assessment of clinical reasoning in the Olympiad for medical sciences students Iranian Journal of Medical Education 2010 10 5 1056 1067 10.4103/2277-9531.94420 PMC3577397 23555113

