# Supplementary material for: An innovative method to assess clinical reasoning skills: Clinical reasoning tests in the second national medical science Olympiad in Iran
Source: BMC Res Notes. 2011 Oct 17;4:418. doi: 10.1186/1756-0500-4-418 (PMC3215186; doi:10.1186/1756-0500-4-418)
Supplement: Additional file 1 — Appendix. A sample of clinical reasoning tests [file 1756-0500-4-418-S1.DOC]

**Additional files**

**Additional file I: Appendix**

**A sample of clinical reasoning tests**

One example of each clinical reasoning test are mentioned below

**1-Example of a KF test item**

The patient, a 60-year-old male, comes to the emergency room with a chief complaint of vertigo and diplopia. The patient reports a history of hypertension. On physical examination his pupils are reactive and normal.

What is your most probable diagnosis?

With respect to your diagnosis, which elements of his history and physical examination would you particularly want to elicit? (Select up to 4)

A: Horizontal nystagmus

B: Vertical nystagmus

C: Nausea and vomiting

D: Increased vertigo on Valsalva maneuver

E: Gait disturbance

F: Positional vertigo

G: Tinnitus

H: Hearing loss

I: Slurred speech

J: Response to Antihistamines

K: Fever

L: History of trauma

M: History of ear infection

N: History of drug use

O: Improvement on Dix-Halpike maneuver

**2-Example of an SCT item**

A 65-year-old male comes to the emergency room with chief complaint of hematuria. He has a history of diabetes mellitus and hypertension from 10 years ago and diarrhea from 3 days ago.

Serum creatinine: 2.5 mg/dL

| This hypothesis would become | And then you were to find | If you are thinking of |
| --- | --- | --- |
| -2 -1 0 +1 +2 | Improvement of symptoms by prednisone | Acute renal failure with intrinsic causes |
| -2 -1 0 +1 +2 | Lower leg edema | Prerenal azothemia |
| -2 -1 0 +1 +2 | History of vomiting | Aminoglycoside poisoning |

**Scoring key:**

-2 Ruled out or almost ruled out

-1 Less probable

0 Neither less or more probable

+1 More probable

+2 Certain or almost certain

**3-Example of a CRP test item**

The patient is a 46-year-old diabetic man who comes to the emergency room with chest pain since 2 hours prior to admission that lasts for 20 minutes. Now he has no pain and his EKG is normal. His blood sugar is well controlled with glybenclamide. Past history is significant for 10 pack/year smoking and chest pain after walking. His pain was reduced with TNG and warm liquids. This time he experienced pain after a heavy meal diet and the pain was not improved by hot liquids.

1) What do you think the most likely diagnosis is for this patient?

A: Stable angina

B: Pleurisy

C: Esophageal spasm

D: Spontaneous pneumothorax

E: Biliary colic

F: Unstable angina

2) Please list the features of the case which you consider to support your diagnosis and those which oppose it, giving an appropriate sign [positive (+) or negative (-)].

| Supports (+) or opposes (-) | Features |
| --- | --- |

3) If this diagnosis proved incorrect, what would your next choice be?

A: Stable angina

B: Pleurisy

C: Esophageal spasm

D: Spontaneous pneumothorax

E: Biliary colic

F: Unstable angina

4) Please list the features of the case which you consider to support your diagnosis and those which oppose it, giving an appropriate sign [positive (+) or negative (-) ].

| Supports (+) or opposes (-) | Features |
| --- | --- |

**4- Example of a CIP test item**

**History**

1- A 30-year-old female with joint pain

2- A 20-year-old male who recently returned from a trip abroad

3- A 20-year-old male with fatigue and epitasis

4- A 70-year-old male with fever and sever respiratory distress

5- A 50-year-old female with dry cough and dyspnea

6- A 52-year-old female who received heparin prophylaxis

**Past medical history**

1- Not significant

2- History of skin hypersensitivity

3- Not significant except allergy

4- Three-day admission in CCU 1 month ago but after work-up coronary artery disease was ruled out

5- History of COPD and diabetes mellitus

6- History of bloody diarrhea

**Physical examination**

1- Diffuse ecchymosis, normal spleen, no lymphadenopathy, harsh breathing sounds in lower quadrant of right lung

2- Swelling and warmth in right upper extremity

3- Petechiae in extremities, normal spleen, no lymphadenopathy, pale conjunctiva

4- Diffuse expirational wheezing, no fever

5- Petechiae in extremities, splenomegaly and lymphadenopathy

6- No arthritis, butterfly rash on face

1- CIP answer sheet

| ANA= negative | Hb=6 g/dL | WBC=6000/mm^3^ | BUN=35 mg/dL |
| --- | --- | --- | --- |
| CH50= normal | C_4_= normal | C_3_= normal | Cr=2 mg/dL |
|  | PTT=32" | PT=12" | PLT=25000/mm^3^ |

2-

| ANA= negative | Hb=13 g/dL | WBC=8000/mm^3^ | BUN=13 mg/dL |
| --- | --- | --- | --- |
| CH50= normal | C_4_= normal | C_3_=NL | Cr= 0.6 mg/dL |
|  | PTT= normal | PT=normal | PLT=30000/mm^3^ |

3-

| PT=18" | Hb=14 g/dL | WBC=30000/mm^3^ | BUN=30 mg/dL |
| --- | --- | --- | --- |
| PTT=50" | PLT=50000/mm^3^ | C_3_= normal | Cr=1.8 mg/dL |

4-

| PLT=10000/mm^3^ | ANA= negative | Hb=13 g/dL | WBC=100000/mm^3^ | BUN=30 mg/dL |
| --- | --- | --- | --- | --- |
| PT=12",PTT=35" | CH50= normal | C_4_= normal | C_3_= normal | Cr=1.6 mg/dL |

5-

| PTT= normal | Hb=10 g/dL | WBC=6000/mm^3^ | BUN=14 mg/dL |
| --- | --- | --- | --- |
| ANA= negative | C_4_= normal | C_3_= normal | Cr= .6 |
|  |  | PT=normal  CH50=normal | PLT=30000/mm^3^ |

6-

| ANA= + | Hb=11 g/dL | WBC=3000/mm^3^ | BUN=40 mg/dL |
| --- | --- | --- | --- |
|  | C_4_= Low | CH50= Low | Cr= 2.5 mg/dL |
|  | PTT= 30" | PT=12" | PLT=50000/mm^3^ |

| Laboratory data | Physical examination | Past medical history | History |  |
| --- | --- | --- | --- | --- |
|  |  |  |  | A |
|  |  |  |  | B |
|  |  |  |  | C |
|  |  |  |  | D |
|  |  |  |  | E |
|  |  |  |  | F |
